# New-Onset Atrial Fibrillation and Accelerated Kidney Function Decline in Working-Age Adults

**DOI:** 10.1001/jamanetworkopen.2026.12823

**Published:** 2026-05-14

**Authors:** Yuichiro Mori, Keita Hirano, Tatsuyoshi Ikenoue, Arisa Kobayashi, Motoko Yanagita, Shingo Fukuma

**Affiliations:** 1 Department of Human Health Sciences, Graduate School of Medicine, Kyoto University, Kyoto, Japan; 2 Department of Public Health, Faculty of Medicine, Mizayaki University, Mizayaki, Japan; 3 Division of Nephrology and Hypertension, Department of Internal Medicine, The Jikei University School of Medicine, Tokyo, Japan; 4 Department of Nephrology, Graduate School of Medicine, Kyoto University, Kyoto, Japan; 5 Institute for the Advanced Study of Human Biology, Kyoto University, Kyoto, Japan; 6 Department of Epidemiology, Disease Control, and Prevention, Hiroshima University Graduate school of Biomedical and Health Sciences, Hiroshima, Japan

## Abstract

**Importance:**

Early identification of cardiovascular-kidney-metabolic (CKM) syndrome is essential for preventing subsequent organ failure. While atrial fibrillation (AF) is often incidentally detected in working-age adults and such detection is a risk for heart failure development, its specific impact on kidney function decline is yet to be elucidated in this young population.

**Objective:**

To investigate the association of new-onset AF with subsequent kidney function decline in working-age adults.

**Design, Setting, and Participants:**

This retrospective cohort study was conducted using annual health screening records (serial electrocardiogram and estimated glomerular filtration rate [eGFR]) and insurance claims data from April 1, 2015, to March 31, 2023, in Japan. Participants included screening attendees aged 35 to 59 years (working-age population) in sinus rhythm without prior AF, cardiovascular comorbidities, or end-stage kidney disease. Data were analyzed from April 1, 2024, to November 1, 2025.

**Exposure:**

Individuals who developed new-onset AF during the annual screening interval were matched 1:5 to those who did not.

**Main Outcomes:**

The primary outcome was annual rate of eGFR decline, estimated using linear mixed-effects models. The secondary outcome was a 30% or greater decline in eGFR from baseline, assessed using a Cox proportional hazards model.

**Results:**

Among 7 717 526 individuals with sinus rhythm at baseline, 141 060 adults were matched (mean [SD] age, 49.8 [6.6] years; 81.8% male; median [IQR] follow-up, 4.73 [3.50-6.22] years). New-onset AF was associated with a greater annual rate of eGFR decline (−1.23 [95% CI, −1.26 to −1.21] mL/min/1.73 m^2^) than that of individuals who did not develop AF (−0.94 [95% CI, −0.95 to −0.93] mL/min/1.73 m^2^), with a difference of −0.29 (95% CI, −0.32 to −0.26) mL/min/1.73 m^2^ (*P* < .001). Also, new-onset AF was associated with an increased incidence of a 30% or greater eGFR decline (hazard ratio, 2.91; 95% CI, 2.72-3.11; *P* < .001).

**Conclusions and Relevance:**

In this retrospective cohort study of working-age adults, new-onset AF was associated with an acceleration in kidney function decline. Further investigation is warranted to clarify the impact of AF burden on the decline and to determine the effectiveness of AF treatments for improving kidney outcomes.

## Key Points

**Question:**

Is new-onset atrial fibrillation associated with accelerated kidney function decline in working-age adults?

**Findings:**

In this cohort study of 23 510 adults with new-onset atrial fibrillation matched to 117 550 controls from a nationwide Japanese database, estimated glomerular filtration rate declined by 1.23 mL/min/1.73 m^2^ annually in the individuals with new-onset atrial fibrillation and by 0.94 mL/min/1.73 m^2^ in the control group (a statistically significant difference of 0.29 mL/min/1.73 m^2^).

**Meaning:**

These findings suggest that new-onset atrial fibrillation may accelerate kidney function decline in working-age adults, underscoring the importance of assessing cardiovascular, kidney, and metabolic health when atrial fibrillation is diagnosed in this population to prevent subsequent organ failure.

## Teaser

This cohort study examines whether the onset of atrial fibrillation in healthy working-age adults is associated with changes in kidney function, using nationwide longitudinal health screening and insurance claims data.

## Introduction

Cardiovascular-kidney-metabolic (CKM) syndrome was defined by the American Heart Association in 2023 to highlight the interplay among metabolic risk factors, chronic kidney disease (CKD), and cardiovascular disease.^1,2^ Identifying CKM syndrome early is essential to interrupt this risk amplification that leads to clinical organ failure.^1^ Atrial fibrillation (AF) is recognized as a common comorbidity of established heart failure and CKD,^3,4^ but it can be detected as an isolated finding in working-age adults. While such incidentally detected AF in a young population is indeed a risk for heart failure development,^5^ whether it is also a risk for kidney function decline, potentially further accelerating the progression of CKM syndrome, remains unclear.^1^

Existing evidence about the relationship between new-onset AF and subsequent kidney function trajectory is limited, especially in the working-age population. Previous investigations focused largely on older, comorbid populations,^6,7^ limiting generalizability to younger and healthier individuals among whom early risk identification is essential. Furthermore, many studies have been limited to identify a clear temporal sequence between AF onset and kidney function decline.^8,9^ While a 2021 Mendelian randomization study suggested a causal link, its design also could not elucidate the temporal trajectory.^10^ Thus, clarifying whether AF onset in this young population precedes subsequent kidney function decline is critically important for early identification and prevention of CKM syndrome.

To address this knowledge gap, this study leveraged a nationwide public health insurer database in Japan, capturing serial annual estimated glomerular filtration rate (eGFR) measurements and electrocardiogram (ECG) results from the working-age general population. This unique opportunity allowed us to ascertain new-onset AF and track subsequent kidney function trajectory. Thereby, we aimed to determine (1) the association of new-onset AF with the subsequent rate of eGFR decline in the working-age general population, and (2) the difference in this trajectory based on subsequent AF persistency. These insights are essential for informing optimal treatment strategies for new-onset AF and mitigating the growing global burden of CKM syndrome.

## Methods

### Study Design and Setting

The institutional review board of Kyoto University Graduate School of Medicine approved all study procedures and waived the requirement for informed consent from individuals due to the use of anonymized data. This retrospective cohort study used insurance claims data and health screening records in the Japan Health Insurance Association (JHIA) database, was conducted in accordance with the Declaration of Helsinki,^11^ and followed the Strengthening the Reporting of Observational Studies in Epidemiology (STROBE) reporting guideline.^12^

Established in 2008, the JHIA serves as Japan’s largest public health insurer, covering more than one-quarter of the working-age population by providing insurance for employees in small- to-mid-sized companies and acting as a pivotal entity in the country’s universal health care coverage system.^13^ This study used insurance claims, death certificates, and health screening records provided by the JHIA for the period spanning April 1, 2015, to March 31, 2023. In Japan, it is obligatory for all social health insurers, including the JHIA, to provide annual health screening for their beneficiaries.^14^ Within insurance coverage by the JHIA, these screenings were fully subsidized for beneficiaries. The screening included physical examination, laboratory testing, self-reported lifestyle information, and 12-lead ECGs.^15^

Patients or the public were not included in the design of the study or recruitment into the study because of the observational nature of the study design. Study results will be disseminated via presentations in academic conferences and press releases.

### Study Population

We first identified individuals aged 35 to 59 years who underwent at least 1 annual health screening between April 1, 2015, and March 31, 2020. Medical histories were ascertained using the *International Classification of Diseases and Related Health Problems, Tenth Revision* (*ICD-10*) codes from insurance claims data, with a lookback period from the date of insurance enrollment up to the health screening date. These *ICD-10* codes were available from insurance enrollment, even before April 1, 2015. The list of *ICD-10* codes used to define each variable is presented in eTable 1 in Supplement 1.

For each annual screening cycle (beginning April 1 and ending March 31), the following exclusion criteria were applied to each screening record (index screening): (1) a lookback period of fewer than 365 days; (2) evidence of AF at the screening or prior, indicated through either health screening ECG (Minnesota code 8-3-1 or 8-3-3) or insurance claims (*ICD-10* code I48); (3) a history of cardiovascular comorbidities, including cardiac disease (*ICD-10* codes B332, I01, I020, I05-I09, I11, I20-I52, or Q20-28), cerebrovascular disease (*ICD-10* codes I60-69), or peripheral vascular disease (*ICD-10* codes K551, K558, K559, I70, I71, I731, I738, I739, I771, I790, I792, Z958, or Z959); (4) an eGFR less than 15 mL/min/1.73 m^2^, 150 mL/min/1.73 m^2^ or greater, or missing data; (5) prior insurance claims of kidney replacement therapy (KRT), including dialysis or kidney transplant; (6) an absence of ECG records in the next screening; (7) adverse cardiovascular events until the next screening, defined as hospitalizations for myocardial infarction (I21 or I22),^16^ stroke (*ICD-10* codes I60-64),^16^ or heart failure (HF; identified by *ICD-10* codes I50, I110, I130, or I132)^16^ (these observations were excluded because they can substantially affect both AF detection and eGFR trajectories, which would disproportionately confound the association); and (8) missing values in covariates used for matching.

### Exposures

**Figure 1.**
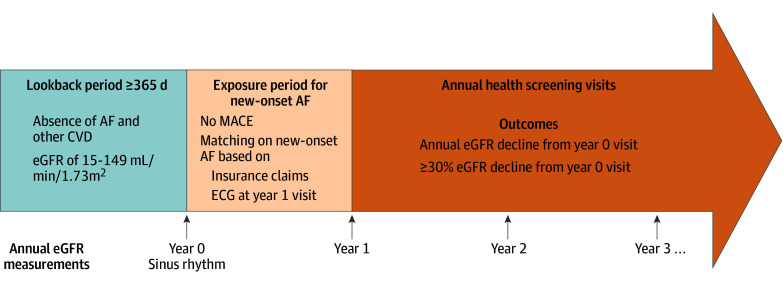
Study Design AF indicates atrial fibrillation; CVD, cardiovascular disease, ECG, electrocardiogram; eGFR, estimated glomerular filtration rate; MACE, major cardiovascular adverse events.

We defined an exposure period for AF identification as the period between the index screening and the screening in the next cycle (Figure 1). New-onset AF was identified based on (1) an ECG performed at the screening in the next cycle, and (2) AF-related outpatient visits during the exposure period, ascertained with insurance claims (*ICD-10* code I48). In the health screenings, AF was defined based on Minnesota codes 8-3-1 or 8-3-3, with physician verification performed according to the established screening program protocol.^17^

### Outcomes and Follow-Up

The primary outcome was the rate of eGFR decline. The secondary outcome was an eGFR decline event, which was defined as a 30% or greater decline in eGFR compared with the index screening, measured at health screenings during follow-up. The eGFR data during follow-up were derived from annual health screenings after the exposure period (ie, screening in year 1 and thereafter), excluding records after initiating KRT. Individuals without new-onset AF in the exposure period who subsequently developed AF during the follow-up period were continuously analyzed as nonexposed. Participants were followed until all-cause mortality events, initiation of KRT, discontinuation of insurance coverage, or the end of the study period (March 31, 2023), whichever occurred first.

### Covariates

Baseline characteristics for age, sex at birth, body mass index (BMI, calculated as weight in kilograms divided by height in meters squared), systolic blood pressure (SBP), diastolic blood pressure (DBP), serum high-density lipoprotein cholesterol, directly measured serum low-density lipoprotein cholesterol (LDL-C), serum creatinine, serum uric acid, hemoglobin, fasting plasma glucose (FPG), hemoglobin A1c (HbA_1c_), dipstick proteinuria, and self-reported current smoking status were collected from the health screening records. The mean of 2 consecutive blood pressure (BP) measurements in a single health screening visit was recorded as the participant’s BP.^18^ Validated oscillometric devices were used by health technicians for BP measurements. Blood samples were analyzed within 24 hours after being centrifuged at each screening facility. Current smoking was defined as having smoked within a month with a smoking duration longer than 6 months, and having consumed more than 100 lifetime cigarettes.^19^ The Japanese eGFR equation^20^ was used to calculate eGFR in accordance with the local society guideline.^21^ Information on a history of hypertension (*ICD-10* codes I11-I15)^22^ and diabetes (*ICD-10 *codes E10-E14)^22^ was collected from insurance claims records during the lookback period. Diabetes status at the index screening was categorized into 3 statuses as follows: (1) nondiabetes, no use of antidiabetic medications, FPG less than 100 mg/dL, and HbA_1c_ less than 5.6%; (2) diabetes, the use of antidiabetic medications, FPG of 126 mg/dL or greater, or HbA_1c_ of 6.5% or greater; and (3) prediabetes, the conditions meeting neither of the criteria for nondiabetes nor diabetes. Because the health screening requires measurements of either FPG or HbA_1c_, diabetes status was defined using the available measure when the other measure was missing.

### Matching

To balance baseline characteristics between individuals who developed new-onset AF and those who did not, we performed propensity score matching. For each individual with new-onset AF, we matched up to 5 individuals in the same screening cycle who did not develop AF during the exposure period. Matching was performed in chronological order without replacements, based on the nearest-neighbor approach and a multivariable logistic regression model to predict AF identification during the exposure period. The matching ratio was determined to maintain statistical precision^23^ and computational efficiency. Matching variables included age, sex, BMI, hypertension, DM status, hemoglobin, HDL-C, LDL-C, eGFR, dipstick proteinuria, uric acid, current smoking, and the duration of the exposure period.

### Statistical Analysis

Data analysis was conducted from April 1, 2024, to November 1, 2025. Means, SDs, numbers, and percentages were used to report descriptive statistics. In the propensity score–matched cohort, the annual rate of eGFR decline was estimated using a linear mixed-effects model with random intercepts and slopes that varied by individual, along with an unstructured covariance structure for these random effects.^24^ A univariable Cox proportional hazards model was used to estimate hazard ratios (HRs) and 95% CIs for the association between new-onset AF and eGFR decline events. In a secondary analysis, for individuals whose AF was initially confirmed through outpatient visits during the exposure period, we compared the annual rate of eGFR decline based on their AF status at the next screening (reconfirmed AF vs sinus rhythm). This comparison used a linear mixed-effects model, which adjusted the covariates used for propensity score matching as fixed effects.

Subgroup analyses on the annual rate of eGFR decline were conducted by sex, baseline proteinuria, and diabetes status. Additionally, to evaluate preexisting between-group differences in eGFR decline, the annual rate of eGFR decline before the index screening was estimated, using available eGFR measurements and the same linear mixed-effects model structure as in the main analysis. We also conducted a sensitivity analysis that repeated the main analysis among individuals with 3 or more eGFR measurements, including the index screening. *P* values were 2-sided, and *P* < .05 was considered statistically significant. Statistical analyses were performed using R, version 4.3.1 (R Foundation).

## Results

### Participant Characteristics

Among 20 969 945 unique individuals aged 35 to 59 years during the study period, 11 679 560 (55.7%) attended at least 1 annual health screening between April 2015 and March 2020, with a total of 33 928 053 person-years of annual health screening records. Among these, 7 717 526 individuals (23 405 909 screening records) with sinus rhythm remained after applying selection criteria. During the exposure period (mean [SD], 370.3 [46.5] days), new-onset AF was identified in 23 803 individuals (eFigure in Supplement 1). After the matching procedures, 23 510 individuals with new-onset AF were matched to 117 550 unique individuals in the same cycles who did not develop new-onset AF during the exposure period. Among the 23 510 matched AF cases, 16 055 (68.3%) were identified during outpatient visits, whereas 7455 (31.7%) were initially identified in an ECG at the next annual screening visit.

Among the 141 060 total matched individuals, the mean (SD) age was 49.8 (6.6) years, 25 614 (18.2%) were female, 115 446 (81.8%) were male, 34 453 (24.4%) had hypertension, 12 775 (9.1%) had diabetes, 9421 (6.7%) had an eGFR lower than 60 mL/min/1.73 m^2^, and 7994 (5.7%) had a dipstick proteinuria of 1+ or greater. The propensity score matching achieved a good matching balance (Table 1). During the follow-up (median [IQR], 4.73 [3.50-6.22] years), the median (IQR) number of eGFR measurements was 5 (4-6), KRT was initiated in 41 of 23 510 individuals (0.2%) with new-onset AF and 125 of 117 550 individuals (0.1%) without, all-cause mortality events occurred in 250 of 23 510 individuals (1.1%) with new-onset AF and 756 of 117 550 individuals (0.6%) without, and AF was newly identified in 1034 of 117 550 individuals (0.8%) in the matched non-AF group.

**Table 1.** Background Characteristics Before and After Matching

Characteristic	Before matching^a^			ASMD	After matching			ASMD
	Overall (N = 23 405 909)	No AF (n = 23 382 106)	New-onset AF (n = 23 803)		Overall (N = 141 060)	No AF (n = 117 550)	New-onset AF (n = 23 510)	
Age, mean (SD), y	46.3 (6.8)	46.3 (6.8)	49.8 (6.6)	0.526	49.8 (6.6)	49.8 (6.5)	49.8 (6.6)	0.006
Sex, No. (%)								
Male	15 145 663 (64.7)	15 126 107 (64.7)	19 556 (82.2)	0.403	115 446 (81.8)	96 159 (81.8)	19 287 (82.0)	0.006
Female	8 260 246 (35.3)	8 255 999 (35.3)	4247 (17.8)		25 614 (18.2)	21 391 (18.2)	4223 (18.0)	
BMI, mean (SD)	23.4 (3.9)	23.4 (3.9)	24.6 (4.4)	0.265	24.5 (4.3)	24.5 (4.3)	24.5 (4.4)	0.004
SBP, mean (SD), mmHg	121.3 (16.7)	121.3 (16.7)	127.8 (18.4)	0.371	127.8 (18.1)	127.8 (18.0)	127.8 (18.4)	<0.001
DBP, mean (SD), mmHg	75.8 (12.3)	75.8 (12.3)	80.4 (13.2)	0.358	80.4 (12.8)	80.4 (12.7)	80.4 (13.2)	0.002
HDL-C, mean (SD), mg/dL	62.6 (17.0)	62.6 (17.0)	60.7 (17.0)	0.112	60.8 (17.2)	60.8 (17.2)	60.7 (17.0)	0.003
LDL-C, mean (SD), mg/dL	124.1 (32.0)	124.2 (32.0)	122.0 (32.6)	0.067	122.0 (31.6)	122.0 (31.4)	122.0 (32.6)	<0.001
Uric acid, mean (SD), mg/dL	5.6 (1.4)	5.6 (1.4)	6.0 (1.4)	0.307	6.0 (1.4)	6.0 (1.4)	6.0 (1.4)	0.006
Hemoglobin, mean (SD), g/dL	14.5 (1.6)	14.5 (1.6)	14.9 (1.4)	0.281	14.9 (1.4)	14.9 (1.4)	14.9 (1.4)	0.004
eGFR, mL/min/1.73 m^2^								
Mean (SD)	80.5 (13.7)	80.5 (13.7)	79.2 (15.4)	0.093	79.2 (14.4)	79.2 (14.2)	79.2 (15.4)	0.002
15-29	9303 (0.0)	9269 (0.0)	34 (0.1)	0.124	132 (0.1)	98 (0.1)	34 (0.1)	0.043
30-59	1 070 514 (4.6)	1 068 770 (4.6)	1744 (7.3)		9289 (6.6)	7576 (6.4)	1713 (7.3)	
60-89	17 152 432 (73.3)	17 135 315 (73.3)	17 117 (71.9)		103 174 (73.1)	86 265 (73.4)	16 909 (71.9)	
≥90	5 173 660 (22.1)	5 168 752 (22.1)	4908 (20.6)		28 465 (20.2)	23 611 (20.1)	4854 (20.6)	
Proteinuria, No. (%)^b^								
–	20 288 883 (86.7)	20 268 928 (86.7)	19 955 (83.8)	0.125	118 594 (84.1)	98 876 (84.1)	19 718 (83.9)	0.009
±	2 331 103 (10.0)	2 328 630 (10.0)	2473 (10.4)		14 472 (10.3)	12 033 (10.2)	2439 (10.4)	
1+	619 501 (2.6)	618 539 (2.6)	962 (4.0)		5688 (4.0)	4736 (4.0)	952 (4.0)	
2+	131 628 (0.6)	131 322 (0.6)	306 (1.3)		1733 (1.2)	1436 (1.2)	297 (1.3)	
3+	34 794 (0.1)	34 687 (0.1)	107 (0.4)		573 (0.4)	469 (0.4)	104 (0.4)	
Current smoking, No. (%)	8 375 661 (35.8)	8 366 373 (35.8)	9288 (39.0)	0.067	54 740 (38.8)	45 579 (38.8)	9161 (39.0)	0.004
Hypertension, No. (%)	2 542 194 (10.9)	2 536 331 (10.8)	5853 (24.6)	0.366	34 453 (24.4)	28 740 (24.4)	5713 (24.3)	0.003
Diabetes status, No. (%)								
None	15 981 562 (68.3)	15 967 973 (68.3)	13 589 (57.2)	0.248	80 719 (57.2)	67 270 (57.2)	13 449 (57.2)	0.001
Prediabetes	6 264 133 (26.8)	6 256 077 (26.8)	8056 (33.8)		47 566 (33.7)	39 628 (33.7)	7938 (33.8)	
Diabetes	1 160 214 (5.0)	1 158 056 (5.0)	2158 (9.1)		12 775 (9.1)	10 652 (9.1)	2123 (9.0)	

Abbreviations: AF, atrial fibrillation; ASMD, absolute standardized mean difference; BMI, body mass index (calculated as weight in kilograms divided by height in meters squared); DBP, diastolic blood pressure; eGFR, estimated glomerular filtration rate; HDL-C, high-density lipoprotein cholesterol; LDL-C, low-density lipoprotein cholesterol; SBP, systolic blood pressure.

^a^ The non-AF cases in prematching includes the records of same individuals in different screening cycles.

^b^ In dipstick proteinuria testing, the symbol* – *represents a negative result for the presence of proteins in urine; *±*, the presence of trace amounts of protein; +1, approximately 30 mg of protein per dL; +2, approximately 100 mg/dL; and +3, approximately 300 mg/dL.

### Association Between New-Onset AF and eGFR Decline

**Figure 2.**
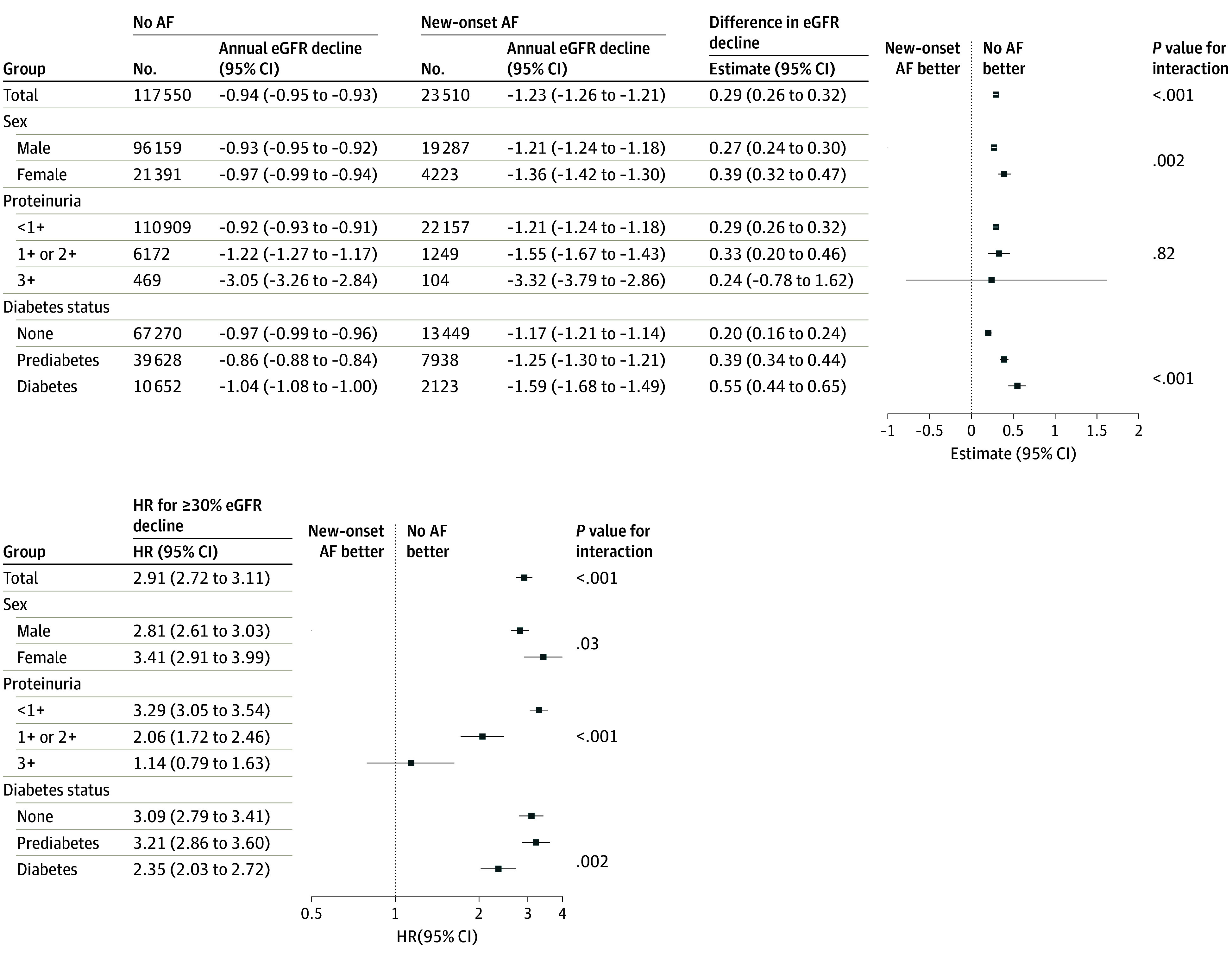
Forest Plots of New-Onset Atrial Fibrillation (AF) and Kidney Function Declines The forest plots display the absolute difference in the annual rate of eGFR decline and the HR for 30% or greater eGFR decline between individuals with and without new-onset AF. All analyses were conducted in the propensity score–matched cohort. For the total row, the *P* value represents the statistical significance of the overall main effect (ie, the overall difference in eGFR slope or the overall HR between the new-onset AF and no AF groups). For the subgroup variables, the* P* value for interaction tests whether the association between new-onset AF and the respective outcome differs significantly across the strata of each subgroup. eGFR indicates estimated glomerular filtration rate; HR, hazard ratio. Error bars represent 95% CIs.

**Figure 3.**
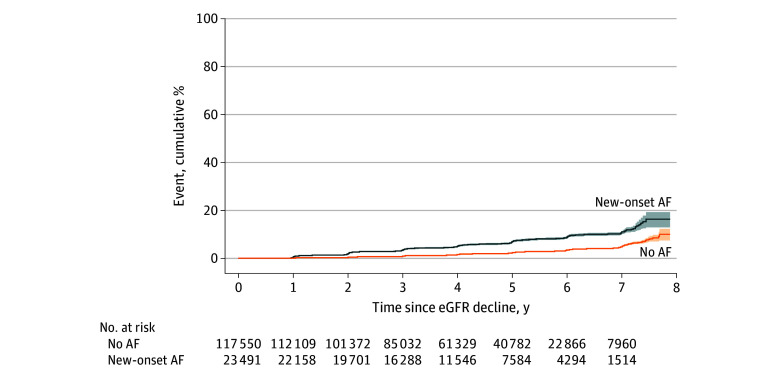
Survival Plot Showing Incidence of 30% or Greater Estimated Glomerular Filtration Rate (eGFR) Decline Events AF indicates atrial fibrillation.

In the matched cohort, the annual rate of eGFR decline was −1.23 (95% CI, −1.26 to −1.21) mL/min/1.73 m^2^ among individuals with new-onset AF and −0.94 (95% CI, −0.95 to −0.93) mL/min/1.73 m^2^ among those without (difference, −0.29 [95% CI, −0.32 to −0.26] mL/min/1.73 m^2^; *P* < .98) (Figure 2). The incidence of a 30% or greater eGFR decline from the index screening was substantially higher in individuals with new-onset AF (HR, 2.91 [95% CI, 2.72-3.11]; *P* < .001) (Figure 3), with estimated 7-year incidence of 11.0% (95% CI, 10.2%-11.7%) among individuals with new-onset AF and 4.9% (4.7%-5.2%) among those without. Conversely, the eGFR decline rate was not significantly different in the period preceding the index screening in the eGFR decline between 2 groups (−0.99 [95% CI, −1.06 to −0.92] mL/min/1.73 m^2^ vs −0.99 [95% CI, −1.02 to −0.96] mL/min/1.73 m^2^; difference, −0.00 [95% CI, −0.08 to 0.07] mL/min/1.73 m^2^; *P* < .001) (eTable 2 in Supplement 1).

In subgroup analyses, the positive association between new-onset AF and accelerated eGFR decline were largely consistent but significantly differed by sex and diabetes status; being female and having diabetes were associated with faster eGFR decline when one had new-onset AF. Conversely, no significant interaction was observed for baseline proteinuria (Figure 2). Among individuals with new-onset AF confirmed in outpatient visits, AF reconfirmation at the subsequent annual screening, compared with returning sinus rhythm, was associated with a greater adjusted annual eGFR decline (−1.55 [95% CI, −1.71 to −1.40] mL/min/1.73 m^2^ vs −1.15 [95% CI, −1.19 to −1.11] mL/min/1.73 m^2^; *P* < .001) (Table 2). A sensitivity analysis restricted to individuals with 3 or more eGFR measurements (127 200 of 141 060 [90.1%]), yielded results consistent with the main analysis (eTable 3 in Supplement 1).

**Table 2.** Annual Rate of Estimated Glomerular Filtration Rate (eGFR) Decline by Clinical Courses of New-Onset Atrial Fibrillation (AF) During the Exposure Period

Clinical course	No.	Annual eGFR decline (95% CI)
AF confirmed during the exposure period	16 055	−1.17 (−1.21 to −1.14)
No AF at the year 1 visit	15 144	−1.15 (−1.19 to −1.11)
AF reconfirmed at the year 1 visit	911	−1.55 (−1.71 to −1.40)
AF confirmed initially at the year 1 visit	7455	−1.42 (−1.48 to −1.36)

## Discussion

In this nationwide cohort study of a Japanese working-age population, new-onset AF was associated with an acceleration in the rate of eGFR decline of 0.29 mL/min/1.73 m^2^ and an increased risk of 30% or greater eGFR reduction. Results were largely consistent across subgroups, with further accelerated decline among females and individuals with diabetes. Furthermore, the persistence of AF at a subsequent screening was associated with a more pronounced eGFR decline.

The present study expands the current state of knowledge on the interplay between new-onset AF and CKM syndrome in working-age adults. Many previous investigations have focused on the association between prevalent AF and CKD^7,8,9^ or on the impact of new-onset AF in older populations with established cardiovascular or kidney disease,^6,7^ thus lacking the granularity to assess kidney function changes after AF onset in a relatively healthy population. The present study, which used a nationwide database of insurance claims and serial health screenings, offered methodological strength, establishing clear temporal sequence across baseline covariates, exposure, and outcome. In addition, the study’s focus on the general working-age population with relatively low prevalence of traditional risk factors for worsening kidney function (eg, hypertension and diabetes) is clinically relevant because early risk identification could provide a substantial window for preventing further disease progression.

The acceleration in eGFR decline after AF identification suggested the independent role of AF in CKD progression in the working-age population. Because the preexisting trajectory in eGFR decline appeared similar between individuals with and without new-onset AF, the greater eGFR decline was likely to be triggered by AF onset. The potential mechanisms linking AF and CKD progression are likely multifactorial. Irregular pulse rhythm and pressure in AF cause greater pressure variability than in individuals in sinus rhythm,^25^ resulting in kidney hypoperfusion and the activation of the renin-angiotensin-aldosterone system.^26,27^ Conversely, the loss of atrial contraction may cause congestion in the right heart and renal vein, which induces congestive nephropathy.^28^ Because incident AF is a strong risk factor for future HF onset,^5^ HF-related biological reactions including systemic inflammation and oxidative stress could also cause chronic kidney injury.^28^

A more pronounced eGFR decline was observed in individuals with reconfirmed AF at a screening visit after their initial identification in an outpatient setting, compared with those who had returned to sinus rhythm. This finding generates the hypothesis that greater eGFR decline is driven by higher AF burden, the clinical importance of which has long been discussed. The greater AF burden is associated with increased risk of adverse CVD outcomes,^29,30,31^ and AF itself is a promoter of an increased AF burden through cardiac electrical remodeling.^32,33^ Although several observational studies have suggested kidney benefits associated with early rhythm control with catheter ablation,^34,35,36^ previous clinical trials have typically not focused on kidney outcomes.^37,38^ Future investigations are warranted to determine whether early rhythm control strategies can attenuate CKD progression in patients with new-onset AF.

### Limitations

The present study has several limitations. First, despite propensity score matching, we cannot fully account for the influence of unmeasured confounders such as detailed measurements (eg, ambulatory BP, medication classes and dosages, glycemic control in DM patients, presence of sleep apnea), socioeconomic status, and lifestyle factors. Second, AF identification was based on annual screening ECGs and AF-related outpatient visits, potentially misclassifying some individuals with paroxysmal and asymptomatic AF to the non-AF group. However, this potential misclassification would likely bias our results toward the null, suggesting the true association might be even stronger. Third, because the present study focused on new-onset AF, whether the observed association is similar or greater in individuals with chronic AF remains unknown. Fourth, data on AF duration or the precise timing of AF onset were not available. However, accurate identification of these factors requires continuous monitoring (eg, by wearable devices), which is likely to come with participant selection bias. Our study may offer one of the best estimates available for detectable AF in this population. Furthermore, while we used a linear mixed-effects model to evaluate the annual eGFR decline, which is a standard methodology as a surrogate of kidney injury,^24^ the limited number of intra-individual measurements precluded complex nonlinear modeling. Future studies with more frequent longitudinal measurements are warranted to more explicitly delineate the trajectory. Fifth, because the study did not include individuals who died during the exposure period, population selection bias exists in our cohort and the results cannot be generalized to acutely ill patients. Sixth, the relatively low proportion of females in the JHIA dataset may restrict the representativeness of our data for Japanese working-age females. Seventh, the results may be influenced by collider stratification bias because we excluded participants who experienced major cardiovascular events during the exposure period. However, this exclusion may bias the results to the null and thus would not substantially change the interpretation of results. Eighth, self-selection bias on health screening attendees (55.7% participation rate in the JHIA during the study period) may exist, which possibly excluded less health-conscious individuals who tend to be older adults and have a lower education attainment level.^39^ Ninth, the study population comprised Japanese citizens, and the findings may not be generalizable to other regions, as AF-related subsequent cardiovascular risk could vary by race, ethnicity, and socioeconomic settings.^39^

## Conclusions

In this nationwide cohort study in working-age adults using insurance claims and annual health screening records in Japan, new-onset AF was associated with an acceleration in eGFR decline. This finding suggests the importance of CKM perspectives in AF management. Further investigation is needed on the cumulative impact of AF on CKD progression and on the effectiveness of AF treatments for improving kidney outcomes.

## Notes

Supplement 1. **eTable 1.** Diagnosis Definitions in the Insurance Claims **eTable 2.** Annual eGFR Decline Prior To Year 0 **eTable 3.** Sensitivity Analysis Among Individuals With ≥3 eGFR Measurements **eFigure.** Patient Selection

Supplement 2.
Data Sharing Statement
