# Supplementary material for: New-Onset Atrial Fibrillation and Accelerated Kidney Function Decline in Working-Age Adults
Source: JAMA Netw Open. 2026 May 14;9(5):e2612823. doi: 10.1001/jamanetworkopen.2026.12823 (PMC13177024; doi:10.1001/jamanetworkopen.2026.12823)
Supplement: Supplement 1. — eTable 1. Diagnosis Definitions in the Insurance Claims eTable 2. Annual eGFR Decline Prior To Year 0 eTable 3. Sensitivity Analysis Among Individuals With ≥3 eGFR Measurements eFigure. Patient Selection [file jamanetwopen-e2612823-s001.pdf]

Supplemental Online Content

Mori Y, Hirano K, Ikenoue T, Kobayashi A, Yanagita M, Fukuma S. New-onset atrial fibrillation and accelerated kidney function decline in working-age adults. *JAMA Netw Open.* 2026;9(5):e2612823. doi:10.1001/jamanetworkopen.2026.12823

eTable 1. Diagnosis Definitions in the Insurance Claims

eTable 2. Annual eGFR Decline Prior to Year 0

eTable 3. Sensitivity Analysis Among Individuals With ≥3 eGFR Measurements

eFigure. Patient Selection

This supplemental material has been provided by the authors to give readers additional information about their work.

© 2026 Mori Y et al. JAMA Network Open.

eTable 1. Diagnosis definitions in the insurance claims

Diagnosis	ICD-10 codes	Reference
Atrial fibrillation	I48	doi: 10.1136/bmj-2023-077209
Hypertension	I11–I15	doi: 10.3820/jjpe.25.e1
Diabetes	E10–E14	doi: 10.3820/jjpe.25.e1
Cardiovascular comorbidities	B332, I01, I020, I05-I09, I11, I20-I52, I60-69, K551, K558, K559, I70, I71, I731, I738, I739, I771, I790, I792, Q20-28, Z958, or Z959	Clinical consensus among the authors[*]

[*]The definition of cardiovascular comorbidities was used as one of the exclusion criteria and was selected based on clinical consensus among the authors, aiming to specifically minimize the inclusion of individuals with significant cardiovascular comorbidities
ICD-10, International Classification of Diseases, 10th Revision

© 2026 Mori Y et al. JAMA Network Open.

eTable 2. Annual eGFR decline prior to Year 0

Measurements and groups	Number of individuals with available measurements	Annual eGFR decline (95%CI)
Health screenings prior to Year 0		
No AF	86,221	-0.99 (-1.02 to -0.96)
New-onset AF	17,109	-0.99 (-1.06 to -0.92)
Health screenings after Year 0		
No AF	117,550	-0.94 (-0.96 to -0.93)
New-onset AF	23,510	-1.23 (-1.26 to -1.21)

AF, atrial fibrillation; eGFR, CI, confidence interval; eGFR, estimated glomerular filtration rate

© 2026 Mori Y et al. JAMA Network Open.

eTable 3. Sensitivity analysis among individuals with ≥ 3 eGFR measurements

Groups	n	Annual eGFR decline (95%CI)
Individuals with three or more measurements		
No AF	106,226	-0.94 (-0.95 to -0.93)
New-onset AF	20,974	-1.22 (-1.25 to -1.20)
Total cohort		
No AF	117,550	-0.94 (-0.96 to -0.93)
New-onset AF	23,510	-1.23 (-1.26 to -1.21)

AF, atrial fibrillation; eGFR, CI, confidence interval; eGFR, estimated glomerular filtration rate

© 2026 Mori Y et al. JAMA Network Open.

eFigure. Patient selection

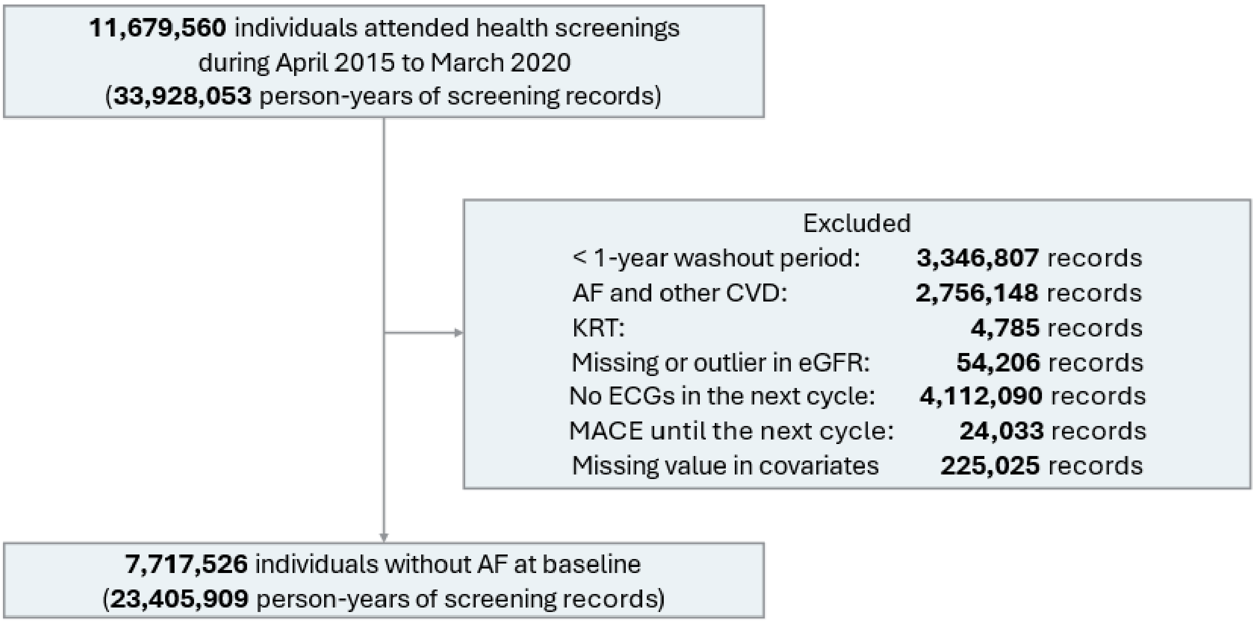

AF, atrial fibrillation; CVD, cardiovascular disease, ECG, electrocardiogram; eGFR, eGFR, estimated glomerular filtration rate

© 2026 Mori Y et al. JAMA Network Open.
