# Supplementary material for: New-Onset Atrial Fibrillation and Accelerated Kidney Function Decline in Working-Age Adults
Source: JAMA Netw Open. 2026 May 14;9(5):e2612823. doi: 10.1001/jamanetworkopen.2026.12823 (PMC13177024; doi:10.1001/jamanetworkopen.2026.12823)
Supplement: Supplement 2. — Data Sharing Statement [file jamanetwopen-e2612823-s002.pdf]

Data Sharing Statement

Mori. New-Onset Atrial Fibrillation and Accelerated Kidney Function Decline in Working-Age Adults. *JAMA Netw Open*. Published May 14, 2026. doi:10.1001/jamanetworkopen.2026.12823

Data
Data available: No
